# *Calbindin-D28k* in the Brain Influences the Expression of Cellular Prion Protein

**DOI:** 10.1155/2018/4670210

**Published:** 2018-02-06

**Authors:** Yeong-Min Yoo, Eui-Bae Jeung

**Affiliations:** ^1^Institute of Forest Science, Department of Forest Environment Protection, College of Forest and Environmental Sciences, Kangwon National University, Chuncheon, 24341 Gangwon-do, Republic of Korea; ^2^Laboratory of Veterinary Biochemistry and Molecular Biology, College of Veterinary Medicine, Chungbuk National University, Cheongju, 28644 Chungbuk, Republic of Korea

## Abstract

The phenotypes of *calbindin-D9k*- (*CaBP-9k*-) knockout (KO), *calbindin*-*D28k*- (*CaBP-28k*-) KO, and *CaBP-9k/28k*-KO mice are similar to those of wild-type (WT) mice due to the compensatory action of other calcium transport proteins. In this study, we investigated the expression of cellular prion protein (PrP^C^) in the brains of *CaBP-9k*-, *CaBP-28k*-, and *CaBP-9k/28k*-KO mice. PrP^C^ expression was significantly upregulated in the brain of all three strains. Levels of phospho-Akt (Ser473) and phospho-Bad (Ser136) were significantly elevated, but those of phospho-ERK and phospho-Bad (Ser155 and 112) were significantly reduced in the brains of *CaBP-9k*-, *CaBP-28k*-, and *CaBP-9k/28k*-KO mice. The expressions of the Bcl-2, p53, Bax, Cu/Zn-SOD, and Mn-SOD proteins were decreased in the brains of all KO mice. Expression of the endoplasmic reticulum marker protein BiP/GRP78 was decreased, and that of the CHOP protein was increased in the brains of those KO mice. To identify the roles of *CaBP-28k*, we transfected PC12 cells with siRNA for *CaBP-28k* and found increased expression of the PrP^C^ protein compared to the levels in control cells. These results suggest that *CaBP-28k* expression may regulate PrP^C^ protein expression and these mice may be vulnerable to the influence of prion disease.

## 1. Introduction

The human cellular prion protein (PrP^C^) is a glycosylphosphatidylinositol- (GPI-) anchored membrane glycoprotein, and a conformationally altered *β*-structure-rich insoluble isoform of PrP^Sc^, scrapie, is an infectious agent responsible for transmissible spongiform encephalopathies, which affect both humans and animals. The activities of PrP^C^ play an important role in the protection against apoptotic and oxidative stress, cellular uptake or binding of copper ions, transmembrane signaling, formation and maintenance of synapses, and adhesion to the extracellular matrix [1, 2].

Calbindin proteins are vitamin D-dependent calcium-binding proteins in the intestine and kidney of chicks and mammals. There are two types: calbindin-D28K (CaBP-28k) and calbindin-D9k (CaBP-9k). CaBP-28k presents in the intestine of birds and in the mammalian kidney. It is also expressed in a number of neuronal and endocrine cells, particularly in the cerebellum. It is encoded in humans by the *CALB1* gene [3]. Calbindin-D9k (CaBP-9k) appears in mammalian intestinal epithelial cells and in the kidney and uterus of some mammalian species. It is encoded in humans by the *S100G* gene, *CALB3* [4]. There is no homology between *CaBP-28k* and *CaBP-9k*, except for their calcium-binding domains (EF hands), of which CaBP-28k has six and CaBP-9k has two [3, 4]. *CaBP-28k* KO in cerebellar Purkinje cells results in distinct cellular and behavioral alterations and marked permanent deficits in motor coordination and sensory processing, suggesting that rapid calcium buffering may directly control behaviorally relevant neuronal signal integration [5]. Lledo et al. [6] demonstrated that *CaBP-28k*-transfected GH3 cells exhibit lower Ca^2+^ entry through voltage-dependent Ca^2+^ channels, resulting in reduced intracellular Ca^2+^ concentrations evoked by voltage depolarization, suggesting that CaBP-28k may protect tissues against Ca^2+^-mediated excitotoxicity. Voigtländer et al. [7] found that a deficiency in *CaBP-28k* in scrapie-infected mice resulted in a significantly lower survival rate, indicating a neuroprotective effect of CaBP-28k.

The phenotypes of *CaBP-9k*-, *CaBP-28k*-, and *CaBP-9k/28k*-KO mice are similar to those of WT mice due to the compensatory action of other calcium transport proteins. Therefore, we investigated the protein expression of PrP^C^ in the brains of *CaBP-28k*-KO mice.

## 2. Experimental Procedures

### 2.1. Animal Experiments

*CaBP-9k*-KO mice were generated as described previously [8], and *CaBP-28k*-KO mice were obtained from the Jackson Laboratory (Bar Harbor, ME, USA). *CaBP-9/28k*-KO mice were generated by breeding *CaBP-9k*-KO female mice with *CaBP-28k*-KO male mice to generate double heterozygotes, which were subsequently bred to obtain homozygous *CaBP-9k/28k*-KO mice. The genotypes of the offspring were determined by genomic PCR analysis, as described previously [9]. Wild-type (WT, C57BL/6), *CaBP-9k*-KO, *CaBP-28k*-KO, and *CaBP-9k/28k*-KO male mice (4 weeks old) were housed in polycarbonate cages and allowed to acclimate to an environmentally controlled room (temperature: 23 ± 2°C; relative humidity: 50 ± 10%, frequent ventilation; and a 12 hr : 12 hr light–dark cycle) before use. Eight male mice of each group (WT, *CaBP-9k* KO, *CaBP-28k* KO, and *CaBP-9k/28k* KO) were used in this study. All animal experimental procedures were approved by the Ethics Committee of Chungbuk National University in the Republic of Korea.

### 2.2. Cell Culture

Rat pheochromocytoma PC12 cells were cultured in Dulbecco's modified Eagle's medium (DMEM; Gibco BRL, Gaithersburg, MD, USA) supplemented with 10% heat-inactivated fetal bovine serum (FBS, Gibco BRL) at 37°C in 5% CO_2_, 95% air in a humidified cell incubator. Knockdown of CaBP-28k with siRNA (Santa Cruz Biotechnology, Santa Cruz, CA, USA) was carried out in PC12 cells by transfection using Lipofectamine™ 2000 (Invitrogen Corporation, CA, USA). Briefly, cells were grown to 30–50% confluence in 100 mm dishes in medium containing 5% FBS without penicillin or streptomycin. Prior to transfection, the medium was replaced with Opti-MEM® (Gibco BRL, CA, USA) for 5 min. Lipofectamine 2000-siRNA complexes were incubated for 20 min at room temperature and then added to the cells, followed by incubation at 37°C in a CO_2_ incubator for 24 hr.

### 2.3. Western Blot Analysis

Mouse total brains were washed twice with ice-cold PBS and then resuspended in 20 mM Tris-HCl buffer (pH 7.4) containing a protease inhibitor mixture (0.1 mM phenylmethylsulfonyl fluoride, 5 *μ*g/mL aprotinin, 5 *μ*g/mL pepstatin A, and 1 *μ*g/mL chymostatin) and phosphatase inhibitors (5 mM Na_3_VO_4_, 5 mM NaF). Whole cell lysates were prepared with a Dounce homogenizer using 20 strokes, followed by centrifugation of the lysates at 13,000 ×g for 20 min at 4°C. Protein concentration was determined using the BCA assay (Sigma, St. Louis, CA, USA). Proteins (40 *μ*g) were separated by 12% SDS-PAGE and then transferred onto polyvinylidene difluoride (PVDF) membranes. The membrane was incubated with antibodies directed against the following proteins: CaBP-9k (Santa Cruz Biotechnology), CaBP-28k (Santa Cruz Biotechnology), PrP (Cell Signaling Technology, Beverly, MA, USA), p-Akt and Akt (Cell Signaling Technology), p-Bad (Ser112, Ser155, and Ser136) and Bad (Cell Signaling Technology), p-ERK and ERK (Santa Cruz Biotechnology), Bcl-2 and Bax (Santa Cruz Biotechnology), Cu/Zn- and Mn-SOD (Cell Signaling Technology), GRP78/Bip and CHOP (Cell Signaling Technology), and GAPDH (Assay Designs, Ann Arbor, MI, USA). Membranes were incubated with anti-rabbit or anti-mouse IgG-conjugated horseradish peroxidase secondary antibodies (Santa Cruz Biotechnology) and then with ECL Western blotting reagents (Pierce Biotechnology, Rockford, IL, USA). Immunoreactive proteins were visualized by exposure to the X-ray film. Protein bands were visualized by image scanning, and optical density was measured using ImageJ analysis software (version 1.37; Wayne Rasband, NIH, Bethesda, MD, USA), after the data were corrected by background subtraction and normalized to GAPDH as an internal control.

### 2.4. Statistical Analysis

Significant differences were determined by ANOVA, followed by Tukey's test for multiple comparisons. Analysis was performed with GraphPad Prism v4.0 (GraphPad Software Inc., San Diego, CA, USA). Values are expressed as means ± SD. A *p* value of <0.05 was considered statistically significant.

## 3. Results

### 3.1. The Expression of the CaBP-9k and CaBP-28k Proteins in the Brain

**Figure 1:**
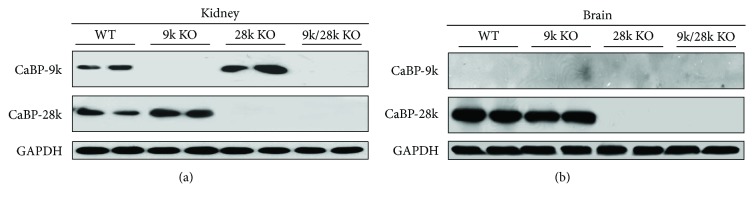
Expression of CaBP-9k and CaBP-28k proteins in the kidneys and brains of *CaBP-9k*-, *CaBP-28k*-, and *CaBP-9k/28k*-KO mice. Expression of CaBP-9k and CaBP-28k proteins in the kidneys (a) and brains (b) of mice was analyzed by Western blot as described in Experimental Procedures. Eight male mice of each group (WT, *CaBP-9k* KO, *CaBP-28k* KO, and *CaBP-9k/28k* KO) were used.

To detect the expression of the CaBP-9k and CaBP-28k proteins in the brain, Western blot analysis was carried out. CaBP-9k protein was expressed only in the kidneys of WT and *CaBP-28k*-KO mice but was detected neither in the kidneys of *CaBP-9k*- or *CaBP-9k/28k*-KO mice nor in the brains of WT, *CaBP-9k*-KO, *CaBP-28k*-KO, or *CaBP-9k/28k*-KO mice (Figure 1). CaBP-28k protein was detected in the kidneys and brains of WT and *CaBP-9k*-KO mice but not in the kidneys or brains of *CaBP-28k*- or *CaBP-9*k*/28k*-KO mice. These results indicate that CaBP-28k protein is expressed in the kidneys and brains of WT and *CaBP-9k*-KO mice but not in those of *CaBP-28k*- or *CaBP-9k/28k*-KO mice.

### 3.2. The Expression of the PrP^C^ Protein in the Brains of KO Mice

**Figure 2:**
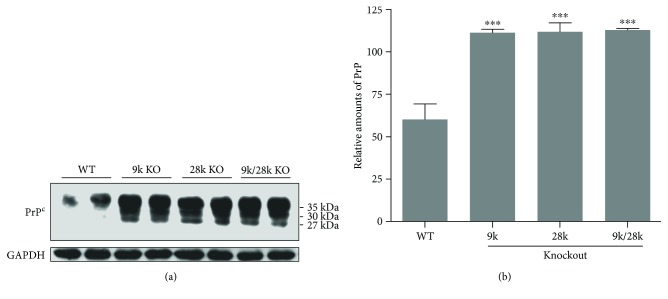
Expression of cellular prion protein (PrP^C^) in the brains of *CaBP-9k*-, *CaBP-28k*-, and *CaBP-9k/28k*-KO mice. Expression of prion protein was analyzed by Western blot (a). The relative amount of PrP^C^ (b) was quantified as described in Experimental Procedures. Values are expressed as means ± SD of eight male mice of each group (WT, *CaBP-9k* KO, and *CaBP-9k/28k* KO). ^∗∗∗^*p* < 0.001 versus wild type (WT).

Next, we investigated the expression of the PrP^C^ protein in the brains of *CaBP-9k*-, *CaBP-28k*-, and *CaBP-9k/28k*-KO mice. PrP^C^ protein was expressed at significantly higher levels in the brains of *CaBP-9k*-, *CaBP-28k*-, and *CaBP-9k/28k*-KO mice than in those of WT mice (Figure 2). These findings indicate that the increase in the PrP^C^ protein in the mouse brains is closely related to the *CaBP-9k*, *CaBP-28k*, and *CaBP-9k/28k* KO.

### 3.3. The Expression Level of p-Akt and p-Bad in the Brains of KO Mice

**Figure 3:**
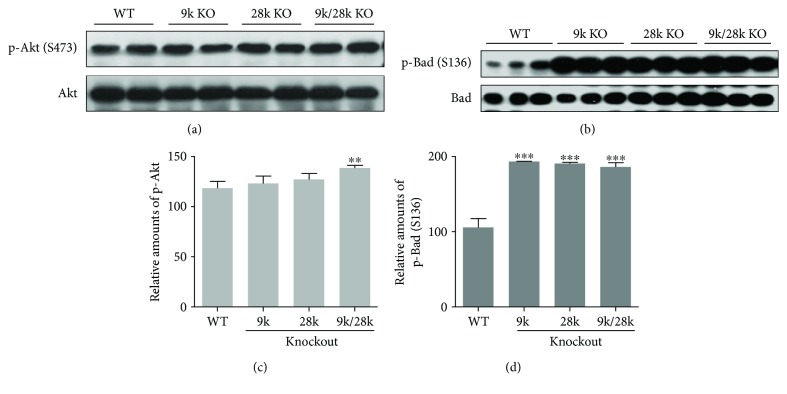
Phosphorylation of Akt and Bad proteins in the brains of *CaBP-9k*-, *CaBP-28k*-, and *CaBP-9k/28k*-KO mice. Phosphorylation of Akt (Ser473) and Bad (Ser136) proteins was analyzed by Western blot (a, b). The relative amounts of p-Akt (Ser473) (c) and p-Bad (Ser136) (d) were quantified as described in Experimental Procedures. Values are expressed as means ± SD of eight male mice of each group (WT, *CaBP-9k* KO, and *CaBP-9k/28k* KO). ^∗∗^*p* < 0.01 and ^∗∗∗^*p* < 0.001 versus WT.

Akt regulates the expression of prosurvival genes involved in cell survival and apoptosis, and the phosphorylation of Bad (Ser136) translocates from the mitochondrial membrane to the cytosol (Manning and Cantley, 2007). In this study, the level of phospho-Akt (Ser473) was significantly increased in the brains of *CaBP-9k/28k*-KO mice and that of phospho-Bad (Ser136) was significantly elevated in the brains of *CaBP-9k*-, *CaBP-28k*-, and *CaBP-9k/28k*-KO mice (Figure 3). These results indicate that the phosphor-Akt increase may play an important role in the phospho-Bad (Ser136) translocation from the mitochondrial membrane into the cytosol.

### 3.4. The Expression Level of p-ERK and p-Bad in the Brains of KO Mice

**Figure 4:**
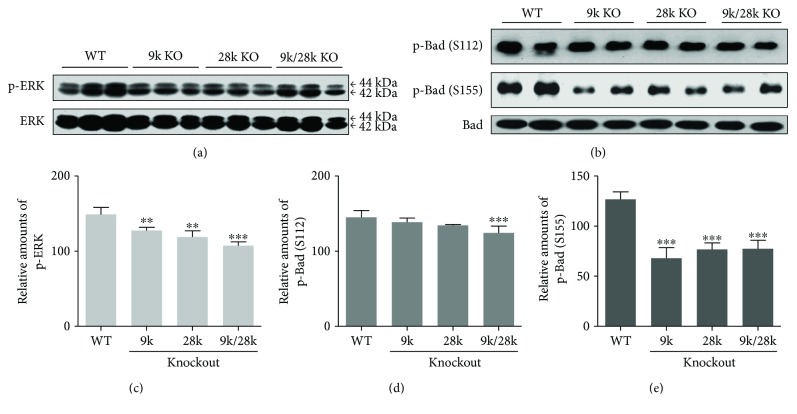
Phosphorylation of ERK and Bad proteins in the brains of *CaBP-9k*-, *CaBP-28k*-, and *CaBP-9k/28k*-KO mice. Phosphorylation of ERK and Bad (Ser122 and 155) proteins was analyzed by Western blot (a, b). The relative amounts of p-ERK (c), p-Bad (Ser122) (d), and p-Bad (Ser155) (e) were quantified as described in Experimental Procedures. Values are expressed as means ± SD of eight male mice of each group (WT, *CaBP-9k* KO, and *CaBP-9k/28k* KO). ^∗∗^*p* < 0.01, and ^∗∗∗^*p* < 0.001 versus WT.

**Figure 5:**
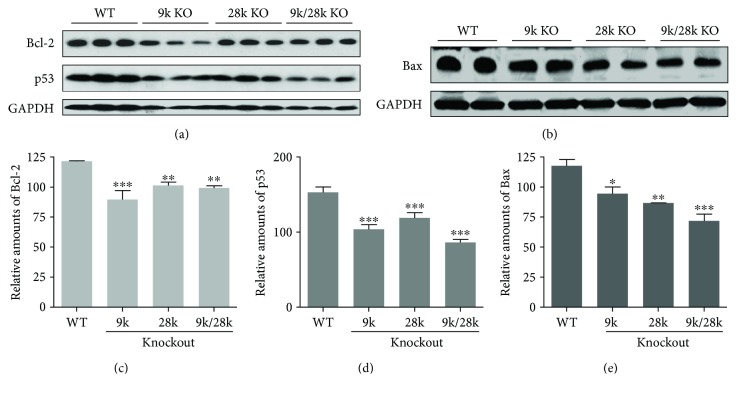
Expression of Bcl-2, p53, and Bax proteins in the brains of *CaBP-9k*-, *CaBP-28k*-, and *CaBP-9k/28k*-KO mice. Expression of Bcl-2, p53, and Bax proteins was analyzed by Western blot (a, b). The relative amounts of Bcl-2 (c), p53 (d), and Bax (e) proteins were quantified as described in Experimental Procedures. Values are expressed as means ± SD of eight male mice of each group (WT, *CaBP-9k* KO, and *CaBP-9k/28k* KO). ^∗^*p* < 0.05, ^∗∗^*p* < 0.01, and ^∗∗∗^*p* < 0.001 versus WT.

The MEK/ERK pathway involves the phosphorylation of Bad on Ser112 and Ser155 in cell growth [10]. In this study, levels of phospho-ERK and phospho-Bad (Ser155 and 112) were significantly reduced in the brains of *CaBP-9k*-, *CaBP-28k*-, and *CaBP-9k/28k*-KO mice (Figure 4). This signal allows Bcl-2 to dissociate from homodimers, resulting in the reduction of antiapoptotic responses. Inactivation of the MEK/ERK cascade can also result in its disassociation from Bcl-2 : Bcl-xL heterodimers, Bax inactivation, and dissociation of Bax : Bax homodimers [9]. Our study demonstrated that expression of the Bcl-2, p53, and Bax proteins is decreased in the brains of *CaBP-9k*-, *CaBP-28k*-, and *CaBP-9k/28k*-KO mice (Figure 5). These findings indicate that phospho-ERK, phospho-Bad (Ser155 and 112), and the expression of the Bcl-2, p53, and Bax proteins are affected by the *CaBP-9k*, *CaBP-28k*, and *CaBP-9k/28k* KO in the mouse brains.

### 3.5. The Expression Level of Cu/Zn-SOD, Mn-SOD, BiP/GRP78, and CHOP Proteins in the Brains of KO Mice

**Figure 6:**
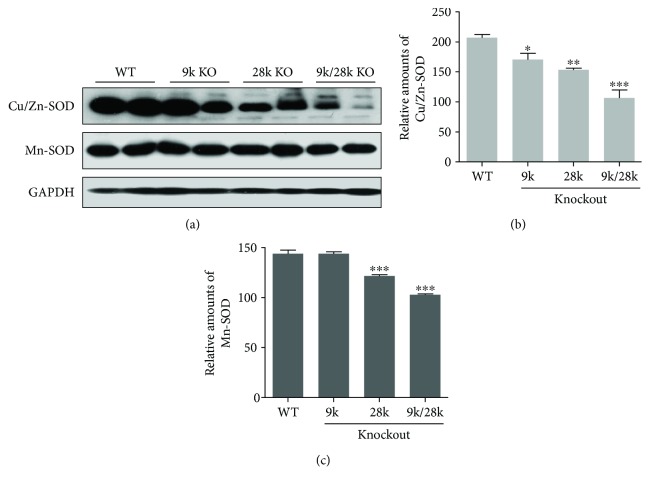
Expression of Cu/Zn-SOD and Mn-SOD proteins in the brains of *CaBP-9k*-, *CaBP-28k*-, and *CaBP-9k/28k*-KO mice. Expression of Cu/Zn-SOD and Mn-SOD proteins was analyzed by Western blot (a). The relative amounts of Cu/Zn-SOD (b) and Mn-SOD (c) proteins were quantified as described in Experimental Procedures. Values are expressed as means ± SD of eight male mice of each group (WT, *CaBP-9k* KO, and *CaBP-9k/28k* KO). ^∗^*p* < 0.05, ^∗∗^*p* < 0.01, and ^∗∗∗^*p* < 0.001 versus WT.

**Figure 7:**
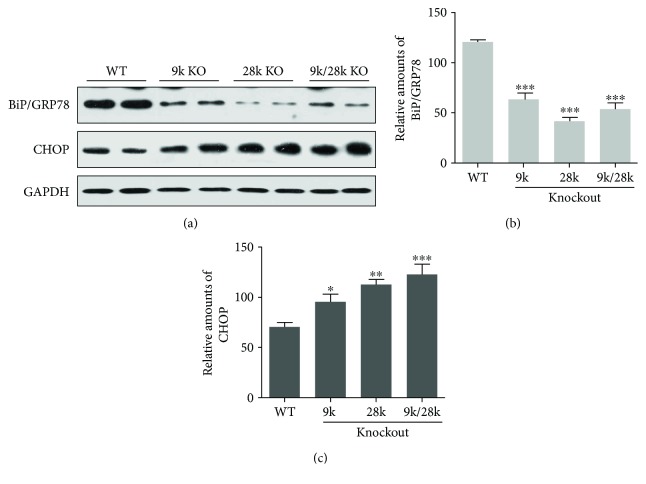
Expression of BiP/GRP78 and CHOP proteins in the brains of *CaBP-9k*-, *CaBP-28k*-, and *CaBP-9k/28k*-KO mice. Expression of BiP/GRP78 and CHOP proteins was analyzed by Western blot (a). The relative amounts of BiP/GRP78 (b) and CHOP (c) proteins were quantified as described in Experimental Procedures. Values are expressed as means ± SD of eight male mice of each group (WT, *CaBP-9k* KO, and *CaBP-9k/28k* KO). ^∗^*p* < 0.05, ^∗∗^*p* < 0.01, and ^∗∗∗^*p* < 0.001 versus WT.

Protein levels of the antioxidant enzyme Cu/Zn-superoxide dismutase (SOD) were decreased in the brains of *CaBP-9k*-, *CaBP-28k*-, and *CaBP-9k/28k*-KO mice, and Mn-SOD protein levels were decreased in the brains of *CaBP-28k*- and *CaBP-9k/28k*-KO mice (Figure 6). Levels of the endoplasmic reticulum stress marker BiP/GRP78 were decreased, and those of the CHOP protein were increased in the brains of *CaBP-9k*-, *CaBP-28k*-, and *CaBP-9k/28k*-KO mice (Figure 7). These results suggest that the expressions of Cu/Zn-SOD, Mn-SOD, BiP/GRP78, and CHOP proteins are closely related to the brains of *CaBP-9k*-, *CaBP-28k*-, and *CaBP-9k/28k*-KO mice.

### 3.6. The Role of CaBP-28k in PC12 Cells

**Figure 8:**
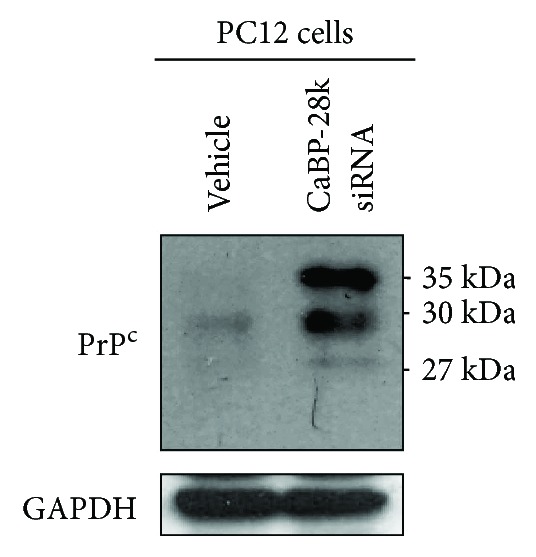
Protein expression of PrP^C^ in GH3 cells and PC12 cells. Rat pituitary GH3 cells and pheochromocytoma PC12 cells were cultured in DMEM supplemented with 10% heat-inactivated FBS at 37°C in 5% CO_2_, 95% air in a humidified cell incubator. To investigate the role of *CaBP-28k* in PC12 cells, cells were transfected with siRNA for *CaBP-28k* using Lipofectamine 2000. Expression of PrP^C^ protein was analyzed by Western blot as described in Experimental Procedures.

PC12 cells expressed CaBP-28k as a sensitive molecular biomarker [10]. To further characterize the role of *CaBP-28k* in rat pheochromocytoma PC12 cells, cells were transfected with siRNA for *CaBP-28k*. Transfection of PC12 cells with siRNA for *CaBP-28k* increased the expression of the PrP^C^ protein relative to controls (Figure 8). This finding indicates that the protein reduction of *CaBP-28k* can increase the PrP^C^ protein.

## 4. Discussion

In this study, CaBP-9k protein was found to be expressed only in the kidneys of WT and *CaBP-28k*-KO mice but was never detected in the brains of any mice, including WT, *CaBP-9k*-KO, *CaBP-28k*-KO, and *CaBP-9k/28k*-KO mice (Figure 1). Normal CaBP-9k protein plays a role as a calcium regulator in mammalian intestinal epithelial cells and in the kidney and uterus of some mammalian species [4, 11, 12]. For example, the *CaBP-9k* gene is associated with the compensatory induction of other calcium transporter genes in duodenal epithelial cells [4, 11] and with uterine functions including fetal implantation, calcium homeostasis, and endometrial cell production [12]. CaBP-9k is involved in the regulation of calcium availability in the vicinity of the implanting embryo during the early phase of embryo implantation [12, 14]. In the present study, CaBP-9k protein expression was not detected in the brains of *CaBP-9k*-KO mice, but the expressions of PrP^C^, Akt, ERK, Bad, Bcl-2, p53, Bax, Cu/Zn-SOD, Mn-SOD, BiP/GRP78, and CHOP proteins were altered in the brains of *CaBP-9k*-KO mice relative to the levels in the brains of WT mice. So far, a study about *CaBP-9k* function in the mouse brain has been unknown. Our results suggest that the knockout of the *CaBP-9k* gene, which is not expressed in the mouse brain, may nonetheless influence the expression of cell signaling molecules, including PrP^C^ in the brain.

CaBP-28k protein is expressed in the kidneys and brains of WT and *CaBP-9k*-KO mice but not in those of *CaBP-28k*- or *CaBP-9k/28k*-KO mice (Figure 1). *CaBP-28k* is a marker of neuronal populations in cerebellar Purkinje cells [5, 13, 14]. Barski et al. [5] demonstrated that *CaBP-28k* expressed in Purkinje cells is an essential determinant of normal motor coordination and sensory integration. The effects of Purkinje cell-specific *CaBP-28k* genetic deletion on visual processing may also depend on the sensitivity of extracerebellar brain structures. Because CaBP-28k is a rapid endogenous calcium-buffering protein [15], the impact of *CaBP-28k* deficiency on Ca^2+^ signaling may contribute to behavioral defects [5]. Also, *CaBP-28k* deletion leads to the upregulation of Akt and the reduction in the levels of phospho-ERK and Bcl-2 [16], which is consistent with the results of the present study. Notably, ERK is highly expressed in the adult mammalian central nervous system, and its activation relies on Ca^2+^ influx via the NMDA receptor [17–19], suggesting that *CaBP-28k* depletion may contribute to neuronal deficits and mitochondrial damage, resulting in synaptic damage and subsequent neurodegeneration [20, 21]. Overexpression of CaBP-28k increases neuronal differentiation and neurite growth [22]. In mice infected with the *β*-structure-rich insoluble isoform of PrP^Sc^, a deficiency in *CaBP-28k* leads to a reduced survival rate, indicating a neuroprotective effect of *CaBP-28k* [7]. However, until now, there has been little research into the interaction between PrP and *CaBP-28k*.

PrP^C^ functions in cellular metabolism and regulates the homeostasis of Cu^2+^ ions. Cultured cerebellar cells lacking PrP^C^ exhibit decreased Cu/Zn-SOD activity, suggesting that PrP^C^ may regulate the incorporation of Cu^2+^ into Cu/Zn-SOD protein [23, 24]. Additionally, mouse brains lacking PrP^C^ exhibit decreased SOD and catalase activities, suggesting that the physiological function of PrP^C^ is related to cellular antioxidant defenses [25, 26]. In the present study, PrP^C^ protein expression was significantly increased in the brains of *CaBP-9k*-, *CaBP-28k*-, and *CaBP-9k/28k*-KO mice (Figure 2(a)), whereas levels of Cu/Zn-SOD and Mn-SOD proteins were decreased (Figure 7), suggesting that PrP^C^ protein in the brains of *CaBP-9k*-, *CaBP-28k*-, and *CaBP-9k/28k*-KO mice may function as a cellular antioxidant defense.

Endoplasmic reticulum (ER) dysfunction plays an important part in a range of neurological disorders, including cerebral ischemia, Alzheimer's disease, multiple sclerosis, amyotrophic lateral sclerosis, and prion diseases. In particular, PrP- and A*β*-induced perturbations of Ca^2+^ homeostasis in the ER are involved in the neuronal loss that occurs in prion diseases and AD [27–29]. The unfolded protein response leads to the upregulation of ER molecular chaperones; the most abundant of which is BiP/GRP78 [30]. Suppression of BiP/GRP78 enhances apoptosis in hippocampal neurons exposed to excitotoxic and oxidative insults [31], whereas its overexpression in primary astrocyte cultures is protective against oxygen and glucose deprivation [32]. Indeed, decreased expression of BiP/GRP78 is associated with neuronal cell death [33, 34]. CHOP is induced after forebrain ischemia in various rodent models, including bilateral common carotid artery [35, 36] or middle cerebral artery occlusion in mice [37] and global cerebral ischemia in rats [38]. The location of CHOP induction in the brain seems to be closely associated with subsequent cell death [38, 39], and deletion of CHOP protects mice during bilateral common carotid artery occlusion [36]. Similarly, depletion of CHOP via RNA interference partially prevents the death of astrocyte cultures stressed by oxygen and glucose deprivation [13]. In the present study, BiP/GRP78 was decreased and CHOP protein was increased in the brains of *CaBP-9k*-, *CaBP-28k*-, and *CaBP-9k/28k*-KO mice (Figure 8), suggesting that these mice may be vulnerable to ER stress and that these genes may influence the expression of PrP^C^ protein.

## 5. Conclusions

Our study demonstrated that the level of PrP^C^ protein was significantly increased in the brains of *CaBP-28k*-KO mice, indicating that *CaBP-28k* expression may regulate PrP^C^ protein expression and these mice may be vulnerable to the influence of prion disease and serve as models in studies of PrP.

## References

[1] Martins V. R., Mercadante A. F., Cabral A. L., Freitas A. R., Castro R. M. (2001). Insights into the physiological function of cellular prion protein. *Brazilian Journal of Medical and Biological Research*.

[2] Westergard L., Christensen H. M., Harris D. A. (2007). The cellular prion protein (PrP^C^): its physiological function and role in disease. *Biochimica et Biophysica Acta (BBA) - Molecular Basis of Disease*.

[3] Kojetin D. J., Venters R. A., Kordys D. R., Thompson R. J., Kumar R., Cavanagh J. (2006). Structure, binding interface and hydrophobic transitions of Ca^2+^-loaded calbindin-D_28K_. *Nature Structural & Molecular Biology*.

[4] Barley N. F., Prathalingam S. R., Zhi P., Legon S., Howard A., Walters J. R. (1999). Factors involved in the duodenal expression of the human calbindin-D9k gene. *The Biochemical Journal*.

[5] Barski J. J., Hartmann J., Rose C. R. (2003). Calbindin in cerebellar Purkinje cells is a critical determinant of the precision of motor coordination. *The Journal of Neuroscience*.

[6] Lledo P. M., Somasundaram B., Morton A. J., Emson P. C., Mason W. T. (1992). Stable transfection of calbindin-D_28k_ into the GH3 cell line alters calcium currents and intracellular calcium homeostasis. *Neuron*.

[7] Voigtländer T., Unterberger U., Guentchev M. (2008). The role of parvalbumin and calbindin D28k in experimental scrapie. *Neuropathology and Applied Neurobiology*.

[8] Lee G. S., Lee K. Y., Choi K. C. (2007). Phenotype of a calbindin-D9k gene knockout is compensated for by the induction of other calcium transporter genes in a mouse model. *Journal of Bone and Mineral Research*.

[9] McCubrey J. A., Steelman L. S., Chappell W. H. (2007). Roles of the Raf/MEK/ERK pathway in cell growth, malignant transformation and drug resistance. *Biochimica et Biophysica Acta (BBA) - Molecular Cell Research*.

[10] Mao Q. Q., Zhong X. M., Feng C. R., Pan A. J., Li Z. Y., Huang Z. (2010). Protective effects of paeoniflorin against glutamate-induced neurotoxicity in PC12 cells via antioxidant mechanisms and Ca^2+^ antagonism. *Cellular and Molecular Neurobiology*.

[11] Choi K. C., An B. S., Yang H., Jeung E. B. (2011). Regulation and molecular mechanisms of calcium transport genes: do they play a role in calcium transport in the uterine endometrium?. *Journal of Physiology and Pharmacology*.

[12] Hong E. J., Jeung E. B. (2013). Biological significance of calbindin-D9k within duodenal epithelium. *International Journal of Molecular Sciences*.

[13] Benavides A., Pastor D., Santos P., Tranque P., Calvo S. (2005). CHOP plays a pivotal role in the astrocyte death induced by oxygen and glucose deprivation. *Glia*.

[14] Girard F., Venail J., Schwaller B., Celio M. R. (2015). The EF-hand Ca^2+^-binding protein super-family: a genome-wide analysis of gene expression patterns in the adult mouse brain. *Neuroscience*.

[15] Maeda H., Ellis-Davies G. C., Ito K., Miyashita Y., Kasai H. (1999). Supralinear Ca^2+^ signaling by cooperative and mobile Ca^2+^ buffering in Purkinje neurons. *Neuron*.

[16] Kook S. Y., Jeong H., Kang M. J. (2014). Crucial role of calbindin-D_28k_ in the pathogenesis of Alzheimer’s disease mouse model. *Cell Death & Differentiation*.

[17] Adams J. P., Sweatt J. D. (2002). Molecular psychology: roles for the ERK MAP kinase cascade in memory. *Annual Review of Pharmacology and Toxicology*.

[18] Bading H., Greenberg M. E. (1991). Stimulation of protein tyrosine phosphorylation by NMDA receptor activation. *Science*.

[19] Fukunaga K., Miyamoto E. (1998). Role of MAP kinase in neurons. *Molecular Neurobiology*.

[20] Barsoum M. J., Yuan H., Gerencser A. A. (2006). Nitric oxide-induced mitochondrial fission is regulated by dynamin-related GTPases in neurons. *The EMBO Journal*.

[21] Li Z., Okamoto K., Hayashi Y., Sheng M. (2004). The importance of dendritic mitochondria in the morphogenesis and plasticity of spines and synapses. *Cell*.

[22] Kim J. H., Lee J. A., Song Y. M. (2005). Overexpression of calbindin-D_28K_ in hippocampal progenitor cells increases neuronal differentiation and neurite outgrowth. *The FASEB Journal*.

[23] Brown D. R., Besinger A. (1998). Prion protein expression and superoxide dismutase activity. *The Biochemical Journal*.

[24] Brown D. R., Schulz-Schaeffer W. J., Schmidt B., Kretzschmar H. A. (1997). Prion protein-deficient cells show altered response to oxidative stress due to decreased SOD-1 activity. *Experimental Neurology*.

[25] Klamt F., Dal-Pizzol F., Conte da Frota M. L. (2001). Imbalance of antioxidant defense in mice lacking cellular prion protein. *Free Radical Biology & Medicine*.

[26] Sauer H., Dagdanova A., Hescheler J., Wartenberg M. (1999). Redox-regulation of intrinsic prion expression in multicellular prostate tumor spheroids. *Free Radical Biology & Medicine*.

[27] Ferreiro E., Resende R., Costa R., Oliveira C. R., Pereira C. M. (2006). An endoplasmic-reticulum-specific apoptotic pathway is involved in prion and amyloid-beta peptides neurotoxicity. *Neurobiology of Disease*.

[28] Ferrer I., Blanco R., Carmona M. (2001). Prion protein expression in senile plaques in Alzheimer’s disease. *Acta Neuropathologica*.

[29] Roussel B. D., Kruppa A. J., Miranda E., Crowther D. C., Lomas D. A., Marciniak S. J. (2013). Endoplasmic reticulum dysfunction in neurological disease. *Lancet Neurology*.

[30] Marciniak S. J., Ron D. (2006). Endoplasmic reticulum stress signaling in disease. *Physiological Reviews*.

[31] Yu Z., Luo H., Fu W., Mattson M. P. (1999). The endoplasmic reticulum stress-responsive protein GRP78 protects neurons against excitotoxicity and apoptosis: suppression of oxidative stress and stabilization of calcium homeostasis. *Experimental Neurology*.

[32] Ouyang Y. B., Xu L. J., Emery J. F., Lee A. S., Giffard R. G. (2011). Overexpressing GRP78 influences Ca^2+^ handling and function of mitochondria in astrocytes after ischemia-like stress. *Mitochondrion*.

[33] Kudo T., Kanemoto S., Hara H. (2008). A molecular chaperone inducer protects neurons from ER stress. *Cell Death & Differentiation*.

[34] Morimoto N., Oida Y., Shimazawa M. (2007). Involvement of endoplasmic reticulum stress after middle cerebral artery occlusion in mice. *Neuroscience*.

[35] Osada N., Kosuge Y., Kihara T., Ishige K., Ito Y. (2009). Apolipoprotein E-deficient mice are more vulnerable to ER stress after transient forebrain ischemia. *Neurochemistry International*.

[36] Tajiri S., Oyadomari S., Yano S. (2004). Ischemia-induced neuronal cell death is mediated by the endoplasmic reticulum stress pathway involving CHOP. *Cell Death & Differentiation*.

[37] Qi X., Okuma Y., Hosoi T., Nomura Y. (2004). Edaravone protects against hypoxia/ischemia-induced endoplasmic reticulum dysfunction. *The Journal of Pharmacology and Experimental Therapeutics*.

[38] Hayashi T., Saito A., Okuno S., Ferrand-Drake M., Dodd R. L., Chan P. H. (2005). Damage to the endoplasmic reticulum and activation of apoptotic machinery by oxidative stress in ischemic neurons. *Journal of Cerebral Blood Flow & Metabolism*.

[39] Oida Y., Shimazawa M., Imaizumi K., Hara H. (2008). Involvement of endoplasmic reticulum stress in the neuronal death induced by transient forebrain ischemia in gerbil. *Neuroscience*.

